# Assessing the Limits of the “Lego-Brick” Approach: Equilibrium Structures of Strained and Flexible Cyclic Molecules

**DOI:** 10.1021/acs.jpca.6c00650

**Published:** 2026-04-15

**Authors:** Silvia Alessandrini, Alessandra Savarese, Mattia Melosso, Luca Bizzocchi, Cristina Puzzarini

**Affiliations:** Dipartimento di Chimica “Giacomo Ciamician”, Università di Bologna, Via P. Gobetti 85, I-40129 Bologna, Italy

## Abstract

An accurate description of molecular structures is essential in several fields of chemistry and, in particular, in high-resolution molecular spectroscopy. The so-called “Lego-brick” approach has proven to provide near-spectroscopic accuracy at a fraction of the computational cost of high-level composite schemes, but its applicability has so far been mainly assessed for rather rigid systems. In this work, we systematically investigate the performance of the “Lego-brick” approach for strained and conformationally flexible cyclic molecules. A chemically diverse benchmark set of three-, four-, and five-membered rings, including heterocycles and species with multiple conformers, is considered. By comparing template-molecule (TM) and full “Lego-brick” (TM+LR) rotational constants with the experimental counterparts, the accuracy of corresponding equilibrium structures is analyzed. The results show that the “Lego-brick” approach retains good accuracy for small cyclic systems, although the data set turned out to be a challenging test case. Linear-regression (LR) corrections are found to be fundamental to achieve the aimed precision. Interestingly, the TM+LR geometries are so accurate that can be employed in the framework of the semiexperimental approach, thus allowing one to obtain equilibrium structures of experimental quality also when there is a lack of isotopic data. Overall, this study delineates the applicability limits of the “Lego-brick” approach for flexible systems, pointing out the ability of significantly improving the initial density functional theory results.

## Graphical abstract

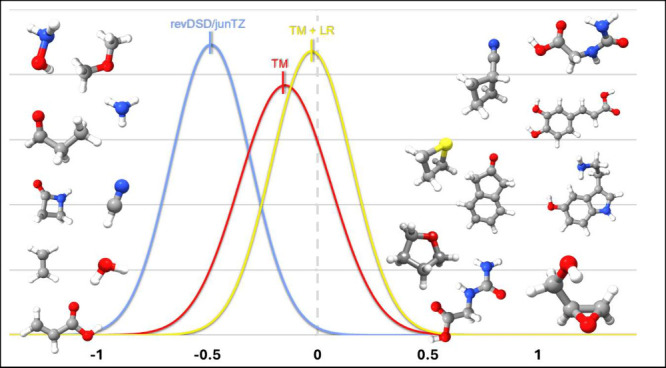

## Introduction

Accurate equilibrium molecular structures are a fundamental prerequisite for the reliable prediction of spectroscopic parameters, in particular rotational constants, which play a central role in high-resolution molecular spectroscopy.^1–3^ State-of-the-art approaches in molecular structure determination using *ab initio* methods rely on composite schemes rooted in the coupled-cluster (CC) theory and its explicitly correlated variant.^4–7^ These approaches can provide a near-spectroscopic accuracy, but at a high computational cost. Indeed, they become rapidly prohibitive as molecular complexity and size increase.^4,5^

In the last years, a fragment-based approach has been proposed in the literature as an alternative to such composite schemes.^8–11^ The strength of this so-called “Lego-brick” approach (also denoted as TM+LR; *vide infra*) is based on the use of density functional theory (DFT), thus making the model scalable to large systems without a significant increase in computational cost.^8,11^ Within the “Lego-brick” approach, the structure of a molecule is constructed using smaller fragments whose equilibrium structures are accurately known, while the linkage parameters between fragments are corrected using linear regression (LR) data.^8–10^ This strategy was found to be very effective in providing an accurate molecular description of medium-to-large rigid molecular systems, such as polycyclic aromatic hydrocarbons (PAHs) and polycyclic aromatic nitrogen heterocycles (PANHs).^12^

The applicability of the TM+LR approach has been extended with success to other chemically relevant species, such as radicals and protonated species.^13,14^ The approach showed no degradation of performance, with statistical uncertainties of about 0.1% for the rotational constants. For radicals, the best approach turned out to be the combination of neutral and open-shell species as fragments,^13^ while for protonated species the corresponding nonprotonated molecules were employed.^14^ Meanwhile, the effectiveness and reliability of the TM+LR approach have been demonstrated several times in the literature for species like cyanopyrenes,^15,16^ ethynylnaphthalenes,^17^ and benzene-derivatives.^18^

The “Lego-brick” approach also represents a solid base for semiexperimental (SE) equilibrium structure determinations. The SE approach combines the use of experimental (vibrational ground state) rotational constants of different isotopologues with computed vibrational corrections in order to derive the equilibrium structure of the molecule via a least-squares fit of the corresponding semiexperimental moments of inertia.^19,20^ Such technique is particularly powerful, but its applicability depends on the quality of the experimental data, the number of isotopically substituted species available and their balance, and, only marginally, on the level of theory adopted for the computation of vibrational corrections.^5,9,10^ Often, a partial determination of the equilibrium structure can only be achieved, and computational estimates have to be employed for the nondeterminable parameters. The TM+LR approach offers a cheap but reliable alternative for such parameters, allowing improvements in SE structure determinations.^21,22^

So far, the “Lego-brick” approach has been benchmarked on rather rigid species, characterized by limited conformational landscapes. In the present work, we aim at extending its application to molecules containing strained rings and/or showing an increased conformational flexibility, also considering a large variety of functional groups. In particular, the benchmark of this study is established on a chemically diverse set of three-, four-, and five-membered rings. These represent a difficult case for the TM+LR approach as they deviate from the typical bonding pattern and challenge the construction based on fragments. In addition to these systems, a greater level of complexity is introduced by moving to larger cyclic species characterized by multiple conformers. This test set is complemented by some rigid molecular systems that act as a bridge between the work of Ye et al.^12^ and the present study. Finally, whenever feasible, the TM+LR approach is used within the SE method to derive accurate equilibrium geometries.

The results of this study will provide new insight into the applicability of the TM+LR approach to a broader chemical space and useful information on the structural features of small rings, that have never been involved in a systematic computational study and the only review reported in the literature dates back to 1980.^23^ This work will also open the way toward the application of the TM+LR approach to open-chain system with one example being also studied.

The following section describes the methodology employed and the test set chosen for the benchmark. Then, the TM+LR results are discussed following an increasing-size order. Semiexperimental equilibrium structures determined in this work will be discussed whenever pertinent.

## Computational Methods

As already mentioned before, the “Lego-brick” approach derives the equilibrium structure of a target molecule using accurate equilibrium structures of smaller fragments, thus employing the so-called template-molecule (TM) approach.^9^ The linkage parameters between fragments are corrected using LR data. For this reason, the “Lego-brick” approach is also denoted as TM+LR in the literature.^12^ The fragments are portions of the target molecule that can be traced back to smaller species with a well-known SE equilibrium structure $\left(r_{e}^{\mathrm{SE}}\right)$ . For example, H_2_O can be used to describe the −OH group, HCCH is the fragment for the —C≡C—H functional group, while formic acid (HCOOH) can be used to describe a carboxyl functionality (−COOH). In the following, a brief outline of the TM and LR strategies is reported, followed by a description of the data set and fragments. All DFT calculations have been carried out using Gaussian16 suite of programs,^24^ and including the D3 dispersion correction^25^ together with the Becke–Johnson (BJ) damping function.^26^

### The “Lego-Brick” Approach

The starting point of the “Lego-brick” approach is the optimization of the target molecule (T) using DFT. Different levels of theory can be employed, but the choice lies usually on the rev-DSDPBEP86 (revDSD) double-hybrid functional^27^ in conjunction with the jun-cc-pV­(T+d)­Z basis set^28^ (where the +d applies to third-row elements). This level, shortly denoted as revDSD/junTZ in the following, is chosen because of the large set of LR parameters already determined in the literature and its consistent performance. The equilibrium structure of the target molecule from revDSD/junTZ $\left(r_{e}^{\mathrm{revDSD}/\mathrm{junTZ},T}\right)$ is improved as follows:

1 $$r_{e}^{\mathrm{TM,T}}=r_{e}^{\mathrm{revDSD}/\mathrm{junTZ},T}+\Delta \mathrm{TM}$$

Here, *r*_*e*_^TM,T^ is the TM-corrected parameter of the target molecule, and ΔTM is the TM-correction based on the fragment, which is expressed as

2 $$\Delta \mathrm{TM}=r_{e}^{\mathrm{SE,F}}-r_{e}^{\mathrm{revDSD,F}}$$

where $r_{e}^{\mathrm{SE,F}}$ is the SE equilibrium parameter of the fragment and $r_{e}^{\mathrm{revDSD,F}}$ is the same parameter computed at the revDSD/junTZ level. Consequently, the improvement provided by the TM approach strongly depends on the quality of the $r_{e}^{\mathrm{SE}}$ structure employed. The underlying idea is that the TM approach is able to transfer the typical accuracy of $r_{e}^{\mathrm{SE}}$ to geometries computed at the DFT level. Clearly, attention should be paid to the reliability of the $r_{e}^{\mathrm{SE}}$ structure and its accuracy which should not be degraded by any bias. In passing we note that the use of any experimental structure is able to improve the DFT ones, as demonstrated (for protonated species) in ref 14 by the so-called LETSGO approach. However, it was also observed that the use of $r_{e}^{\mathrm{SE}}$ geometries significantly lowers the error affecting the rotational constants (used to check the structural accuracy) by a factor of 3: from 0.3% to 0.1%.

To correct the linkage parameters between fragments within the target molecule, the LR coefficients (*a* and *b*) are used. Thus, the LR-corrected parameter is obtained as

3 $$r_{e}^{\mathrm{LR,T}}=\left(1+a\right)r_{e}^{\mathrm{revDSD}/\mathrm{junTZ},T}+b$$

The *a* and *b* values for the revDSD/junTZ level are taken from ref 11 and have been applied to bond distances and valence angles. The combination of the TM and LR corrections provides the TM+LR equilibrium structure of the target molecule, denoted as $r_{e}^{\mathrm{TM}+\mathrm{LR}}$ , while $r_{e}^{\mathrm{TM}}$ will be used to describe the equilibrium structure without LR-corrected parameters. From the $r_{e}^{\mathrm{TM}+\mathrm{LR}}$ and $r_{e}^{\mathrm{TM}}$ geometries, the corresponding equilibrium rotational constants $B_{e}^{\mathrm{TM}+\mathrm{LR},i}$ and $B_{e}^{\mathrm{TM},i}$ , respectively, are straightforwardly derived, where the superscript *i* indicates the inertial axis (*a*, *b* or *c*, so that, e.g., $B_{e}^{i}$ with *i* = *a* corresponds to *A*_*e*_).

To assess the accuracy of the equilibrium structures, rotational constants are compared with the corresponding experimental data in view of their high precision arising from the intrinsic very high resolution of rotational spectroscopy measurements. Experimental rotational constants, the leading term in shaping rotational spectra, refer to the vibrational ground state $\left(B_{0}^{\exp}\right)$ . Thus, a meaningful comparison can be performed only if vibrational effects are incorporated in the computed (TM/TM+LR) equilibrium values. According to second-order vibrational perturbation theory (VPT2),^29^ the vibrational corrections to rotational constants for the vibrational ground state $\left(\Delta B_{0}^{i}\right)$ are defined as

4 $$\Delta B_{0}^{i}=-\frac{1}{2}\underset{r}{\sum }\alpha_{r}^{i}$$

where the sum runs over all the vibrational modes *r* and $\alpha_{r}^{i}$ are the vibration–rotation interaction constants. These latter involve cubic force constants in their expression, thus anharmonic force-field calculations need to be run. Several works in the literature have shown that this term can be safely computed at a low-level of theory,^4^ for example using global-hybrid DFT functionals. For this reason, B3LYP^30,31^ in conjunction with the jun-cc-pVDZ basis set (shortly, B3/junDZ) is employed here for their computation. Overall, the vibrationally averaged rotational constant is computed as

5 $$B_{0}^{\mathrm{theory},i}=B_{e}^{\mathrm{theory},i}+\Delta B_{0}^{B3,i}$$

where theory stands for the level of the equilibrium structure, which is either revDSD/junTZ, TM or TM+LR. To assess the error of $B_{0}^{\mathrm{theory},i}$ in comparison to $B_{0}^{\exp,i}$ , the following statistical quantities are employed:

6 $$\mathrm{\Delta ̅}=\frac{1}{n}\overset{n}{\underset{k=1}{\sum }}\Delta_{k}$$

7 $${\mathrm{\Delta ̅}}_{\mathrm{abs}}=\frac{1}{n}\overset{n}{\underset{k=1}{\sum }}\left|\Delta_{k}\right|$$

$$\Delta_{\max}=\underset{k}{\max}\left|\Delta_{k}\right|$$ 8

9 $$\Delta_{\mathrm{std}}=\sqrt{\frac{1}{n-1}\sum {\left(\Delta_{k}-\mathrm{\Delta ̅}\right)}^{2}}$$

In the equations above, *n* is the number of rotational constants employed in the analysis, while Δ_*k*_ is the deviation (in %) of the computed value with respect to the experimental one. We note that Δ̅ is the mean error and ${\mathrm{\Delta ̅}}_{\mathrm{abs}}$ is the mean absolute error, while Δ_max_ and Δ_std_ are the maximum error and the standard deviation, respectively. To provide an intuitive visualization of these statistical measures, a normal distribution of errors is used (ρ­(Δ)):

10 $$\rho \left(\Delta \right)=\frac{1}{N_{c}}\;\exp{\left[\frac{1}{2}\left(\frac{\Delta_{k}-\mathrm{\Delta ̅}}{\Delta_{\mathrm{std}}}\right)\right]}^{2}$$

where *N*_*c*_ is a normalization constant. From the equation above, it appears that the shift from the *y*-axis of the Gaussian function center represents the mean error, while the width of the Gaussian function is the standard deviation.

### Data Set, Fragments, and Semiexperimental Approach

The data set chosen to assess the performance of the TM+LR approach for flexible ring species is shown in Figure 1 together with the fragments employed. A complete representation showing the different combination of fragments for each molecule is reported in Figures S1–S3 of the Supporting Information (SI). The data set is composed of 50 species, including conformers. Here, we note that conformers and isomers are labeled following the same nomenclature used in the experimental studies referenced. The species considered in this benchmark study can be classified as follows:

- Three-membered ring molecules (**8**): glycidol (*inner* and *outer* conformers),^32^ glycidaldehyde,^33^ methyloxirane,^34^ cyclopropanecarboxaldehyde (*anti* and *syn*),^35^ and N-cyclopropylformamide (*cis* and *trans*).^36^
- Four-membered ring molecules (**10**): oxetane,^37^ trimethylene sulfide,^38^ cyclobutanone,^39^ cyclobutane-1,2-dione,^40^ cyclobutenone,^41^ equatorial-*trans* cyclobutanol,^42^ 2-azetidinone,^43^ cyanocyclobutane,^44^ cyanocyclobutene,^45^ and methylenecyclobutane.^46^
- Five-membered ring molecules (**11**): methylene cyclopentane,^47^ cycloserine I and II,^48^ 2-aminooxazole,^49^ 3-aminoisoxazole,^50^ tetrahydrofuran,^51^ tetrahydrothiophene,^52^ hydantoin,^53^ 2,5-oxazolidinedione,^53^ 2-furonitrile,^54^ and 3-furonitrile.^54^
- Six-membered ring molecules (**12**): toluene,^55^ 3-methylene-1,4-cyclohexadiene,^56^ aspirin (rotamer I and II),^57^ and caffeic acid (*syn* and *anti* isomers, four conformers each).^58^
- Bicyclic compounds (**6**): spiro[2.4]­hepta-4,6-diene,^59^ 2-indanone,^60^ phthalan,^61^ and serotonin (three conformers).^62^

**Figure 1:**
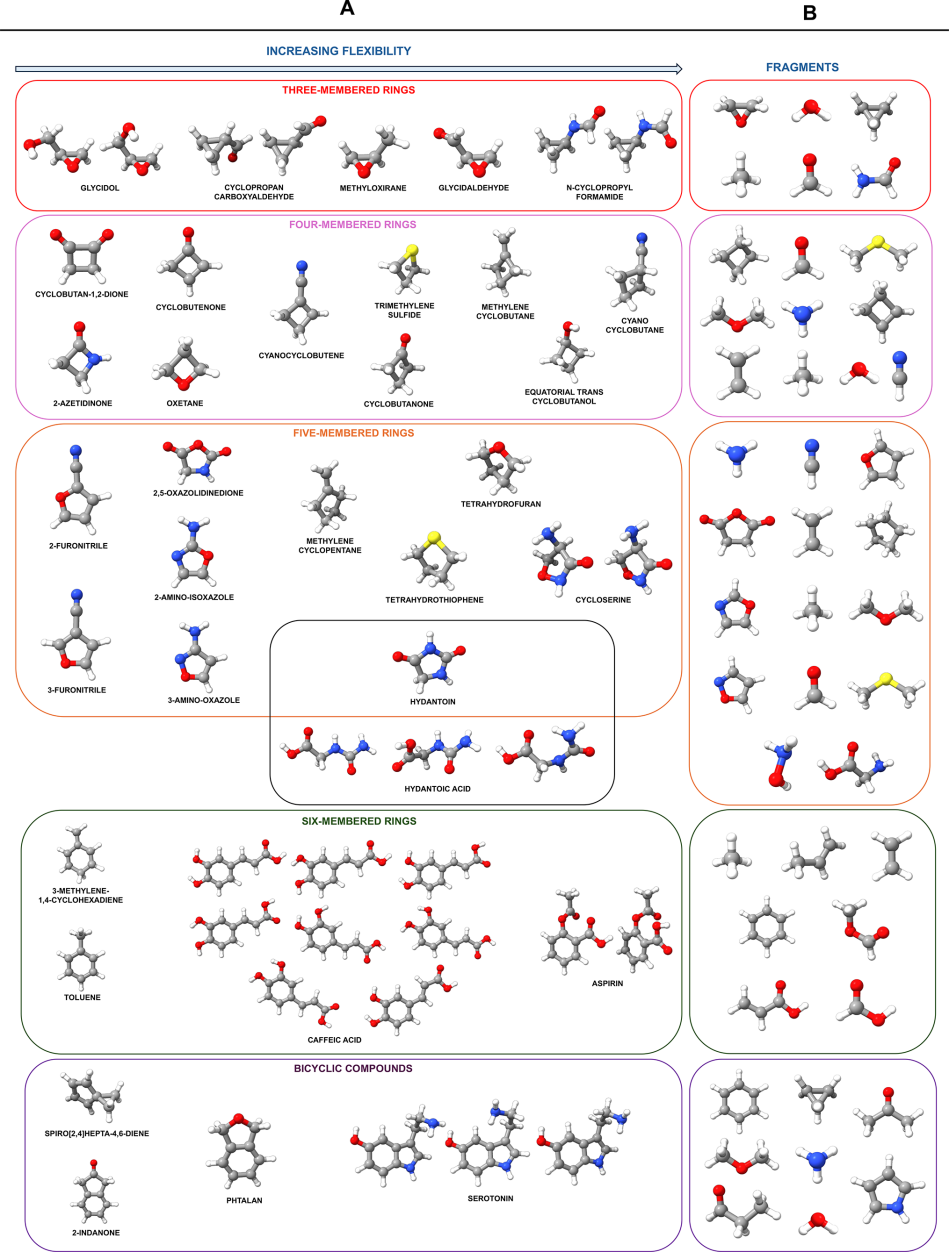
Graphical representation of the molecular species considered in the present study (column A) together with the different fragments employed in the TM+LR approach (column B).

These species are complemented with the inclusion of three conformers of hydantoic acid (C5-I, C5-II, and C7-II),^53^ as these molecules allow for a first inspection of the TM+LR performance when moving from cyclic species (hydantoin and 2,5-oxazolidinedione) to open chain molecules.

From eqs 1 and 2, it is clear that the applicability of the “Lego-brick” approach depends on the availability of SE equilibrium structures for the required fragments. For most of those employed in the present work (see Figure 1), the SE equilibrium geometry was already reported in the literature, in particular in ref 11. The few exception are acetone,^63^ cyclobutane,^64^ methane,^65^ cyclopentane,^66^ cyclopentadiene,^67^ formamide,^68^ and acrylic acid. For the latter, the $r_{e}^{\mathrm{SE}}$ has been purposely derived in the present work using the data from Calabrese et al.^69^ For both forms of acrylic acid (*cis* and *trans*), four different isotopic substitutions other than the parent species were characterized. These are the three ^13^C monosubstituted species and the −COOD isotopologue. As these data do not allow for a full determination of the SE equilibrium structure and some molecular parameters have to be fixed to an accurate estimate, the TM+LR structure was chosen for this purpose (employing as fragments acrolein and water from Ceselin et al.^11^). Similarly to acrylic acid, the TM+LR structure was also employed to derive the partial $r_{e}^{\mathrm{SE}}$ of two target molecules: 2-azetidinone and methylenecyclobutane. For the former, the rotational constants have been experimentally obtained for six isotopologues in ref 43, while for the latter data for five isotopic substitutions were reported in the literature (main species and four ^13^C isotopologues).^42^

## Results

The results are presented in the following based on the size of the ring and using the statistical quantities described in the Computational Methods section. The complete set of structures and equilibrium rotational constants (revDSD/junTZ, TM, and TM+LR) is reported in the SI.

### Three-Membered Rings

The species considered in the data set are either functionalized cyclopropanes or derivatives of oxirane. The statistical quantities for these species are reported in Table 1 and show that, on average, the revDSD/junTZ level of theory predicts the experimental rotational constants with a mean error of 0.69%. The absolute mean error is equal in value to the mean error, thus pointing out that the rotational constants are always underestimated by the revDSD/junTZ level. This deviation is nearly twice that observed for PA­(N)­Hs (0.4%),^12^ but smaller than that obtained for protonated species (0.89%).^14^ The maximum deviation is noted for the rotational constants of *inner* glycidol and is 1.31%. The quantities of Table 1 have been used for building the yellow Gaussian curve of Figure 2.

**Figure 2:**
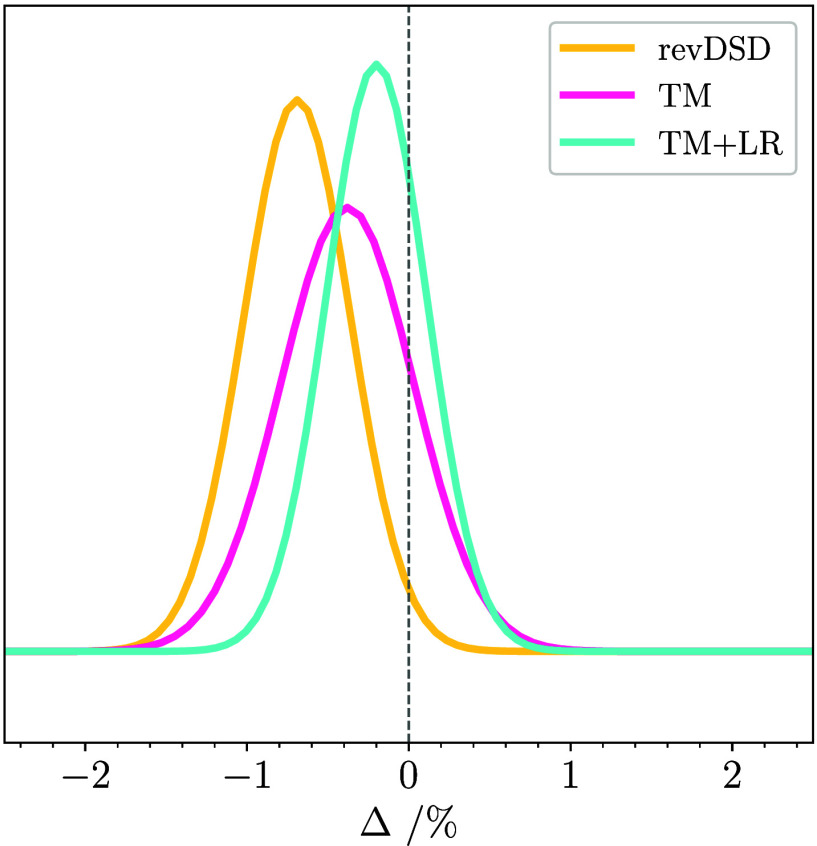
Normal distribution of the errors in the computed rotational constants (*B*_0_^theory,*i*^) in comparison with experimental *B*_0_^exp,*i*^ values for the three-membered ring molecules. The levels of theory are revDSD/junTZ (yellow curve), TM (pink curve), and TM+LR (light-blue curve).

**Table 1:** Statistical Quantities for the Selected Three-Membered Ring Molecules

	Δ̅	Δ̅_abs_	Δ_max_	Δ_std_
revDSD/junTZ	–0.69	0.69	1.31	0.33
TM	–0.37	0.44	1.06	0.40
TM+LR	–0.19	0.30	0.76	0.32

The first step to improve the revDSD/junTZ level is the application of the TM approach (pink curve in Figure 2). The TM rotational constants show a smaller Δ̅ (0.37%), but a larger standard deviation (0.40%). The rotational constants are either under- or overestimated by the TM approach (i.e., no systematic behavior) and the maximum error is only marginally reduced. The incorporation of the LR corrections allows for a good reduction of the statistical uncertainties, with the ${\mathrm{\Delta ̅}}_{\mathrm{abs}}$ being 0.30% and the maximum error 0.76%. Furthermore, the worsening of the standard deviation observed in the TM is recovered, with the Δ_std_ of TM+LR being 0.32%, thus similar to the revDSD/junTZ one.

Overall, as expected,^12^ the TM+LR approach reduces the mean absolute error from 0.7% to 0.3%; however, it is not able to reach, on average, a relative error as small as 0.1%. This is somewhat suspicious because the TM+LR approach was able to successfully reduce the averaged error from 0.9% to 0.1% in the case of protonated species. To understand the reason for such a different behavior, we have carried out a comparison of the TM+LR geometrical parameters of *inner* glycidol with those of a well-determined SE equilibrium structure^70^ and those at the CCSD­(T)/CBS+CV level recently obtained^32^ (we refer the reader to ref 32 and references therein for the definition of the CCSD­(T)/CBS+CV composite scheme and its accuracy). The comparison is shown in Table 2 for both structural parameters and (vibrationally averaged) rotational constants, with the atom labeling being provided in Figure 3. It is noted that the TM+LR approach correctly predicts the bond lengths, with differences within 1–2 mÅ with respect to the other determinations. For valence and dihedral angles, the deviations are always within 1°. However, these differences result in a large variation of the rotational constants. To give an example: TM+LR and CCSD­(T)/CBS+CV rotational constants differ one from the other by 4, 28 and 21 MHz for *A*_0_, *B*_0_ and *C*_0_, respectively. Similar deviations are observed with respect to the SE rotational constants except for the *A*_0_ term, which is predicted very far from the experimental value, with a discrepancy of 43.3 MHz (see Table 2).

**Figure 3:**
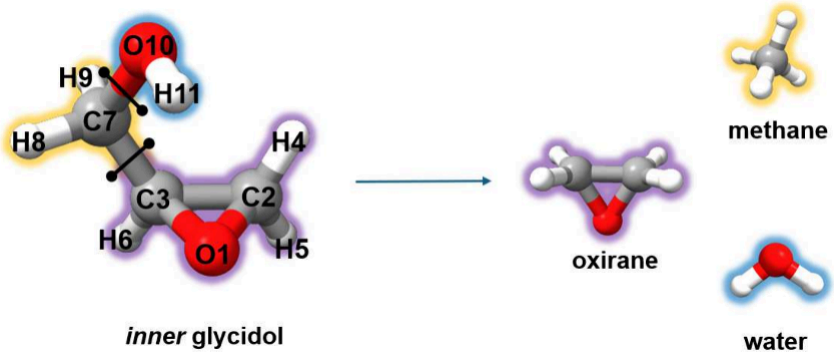
Atom labeling for *inner* glycidol and the fragments employed within the TM approach. The LR-corrected parameters are the C7–C3, C7–O10 distances and the C2C3C7, O10C7C3 angles.

**Table 2:** Structural Parameters and Vibrationally Averaged Rotational Constants of *Inner* Glycidol As Obtained By the TM+LR, CCSD­(T)/CBS+CV and SE Approaches^a^

Parameter	revDSD/junTZ	“Lego-brick”	CCSD(T)/CBS+CV^b^	*r*_*e*_^SE^
		(TM+LR)		ref 70
O1C2	1.4434	1.4392	1.4379	1.44214(64)
O1C3	1.4356	1.4314	1.4299	1.4289(14)
H4C2	1.0837	1.0805	1.0812	1.0807(12)
H5C2	1.0844	1.0812	1.0816	1.0813(12)
H6C3	1.0876	1.0844	1.0851	1.0845(12)
C7C3	1.5096	1.5068	1.5050	1.5064(16)
H8C7	1.0976	1.0942	1.0947	1.0947(12)
H9C7	1.0921	1.0887	1.0895	1.0894(12)
O10C7	1.4135	1.4093	1.4081	1.41019(78)
O10H11	0.9651	0.9614	0.9616	0.9633(19)
C3O1C2	61.00	61.06	61.09	61.184(70)
H4C2C3	119.00	118.90	118.85	118.71(19)
H5C2C3	119.46	119.36	119.43	119.34(18)
H6C3C2	118.47	118.37	118.54	118.47(19)
C7C3C2	121.84	121.81	121.58	121.58(10)
H8C7C3	108.73	108.73	108.73	108.64(19)
H9C7C3	109.77	109.77	109.87	109.83(19)
O10C7C3	112.19	112.11	111.94	112.081(70)
H11O10C7	106.37	106.44	106.43	105.76(18)
H4C2C3O1	–102.88	–102.88	–102.89	–102.97(29)
H5C2C3O1	102.45	102.45	102.48	102.50(30)
H6C3C2O1	–102.79	–102.79	–102.82	–102.71(31)
C7C3C2O1	101.63	101.63	101.40	101.02(13)
H8C7C3C2	–151.15	–151.15	–151.04	–151.30(29)
H9C7C3C2	90.98	90.98	91.01	90.58(29)
O10C7C3C2	–28.03	–28.03	–28.06	–28.48(26)
H11O10C7C3	–50.01	–50.01	–49.50	–48.14(31)

*A*_0_ ^c^ ^,^ ^d^	10313.11 [−0.34%]	10369.30 [0.21%]	10365.33 [0.17%]	10391.16 [0.42%]
*B*_0_ ^c^	4048.54 [−1.31%]	4071.33 [−0.76%]	4099.42 [−0.07%]	4102.65 [0.004%]
*C*_0_ ^c^	3738.26 [−1.15%]	3758.16 [−0.63%]	3779.74 [−0.06%]	3782.80 [0.02%]

a Bond distances in Å, valence and dihedral angles in degrees (°), rotational constants in MHz.

b Taken from ref 32.

c Vibrational corrections included at the B3/junDZ level. The values are Δ*A*_0_ = 70.26 MHz, Δ*B*_0_ = 66.39 MHz, and Δ*C*_0_ = 55.19 MHz.

d In square brackets, deviations with respect to the experimental values of ref 32.

Investigation of how structural parameters affect the rotational constants revealed that the latter are extremely sensible to the C7C3C2O1 dihedral angle. This regulates the position of the carbon of the CH_2_OH group with respect to the ring and can be seen as a dihedral angle connecting the two fragments. In particular, if one considers the TM+LR structure and arbitrarily changes the value of this dihedral angle by 1.5°, the errors on the rotational constants drastically reduce to 0.07%, 0.05% and 0.02% for *A*_0_, *B*_0_ and *C*_0_, respectively. This empirical modification leads to the most accurate equilibrium structure, which is characterized by a nondistorted 3-membered ring, differently to what was claimed in ref 70.

Overall, we expect that dihedral angles are responsible for the slight decrease in the performance of the TM+LR approach with respect to previous investigations. Indeed, incorporation of a LR correction, based on the empirical one applied to the dihedral of *inner* glycidol (*a* = 0.01476, *b* = 0), leads to a reduction of the mean error from ∼0.3% to ∼0.2% for the other oxirane-derivatives considered in the data set. This clearly points out to the need of correction parameters for dihedral angles connecting fragments.

### Four- and Five-Membered Rings

The statistical quantities for four- and five-membered ring molecules are collected in Table 3. Focusing on the first set (four-membered ring), the initial revDSD/junTZ mean error, 0.50%, is reduced to 0.22% by the TM approach. In agreement with what was observed before, the TM+LR brings the error further down, to 0.13%, with an overall reduction of the initial error to about 1/3. The standard deviation of the revDSD/junTZ level is maintained when moving to the TM and TM+LR approaches, and it is about 0.2%.

**Table 3:** Statistical Quantities for the Selected Four- And Five-Membered Ring Molecules

	Δ̅	Δ̅_abs_	Δ_max_	Δ_std_
4-Membered Cycles				
revDSD/junTZ	–0.48	0.50	0.88	0.18
TM	–0.15	0.22	0.47	0.21
TM+LR	–0.03	0.13	0.66	0.18
5-Membered Cycles				
revDSD/junTZ	–0.57	0.57	0.99	0.19
TM	–0.12	0.22	0.58	0.24
TM+LR	–0.01	0.14	0.55	0.19

There are a few interesting cases that deserve to be discussed in more detail. Oxetane and trimethylene sulfide (thiooxetane) are heterocycles with 4 atoms embedding an oxygen or a sulfur atom, respectively. The inclusion of oxygen allows the formation of a regular ring with a *C*_2*v*_ symmetry at equilibrium, while the inclusion of sulfur leads to a distorted ring of *C_s_* symmetry. These molecules represents a nice example of rings that have been templated using noncyclic fragments. Indeed, their TM corrections have been obtained using dimethyl ether or dimethyl sulfide with methane as cofragment. The other case based on open-chain fragments is cyclobutenone, that has been templated using CH_4_, C_2_H_4_, and H_2_CO. For oxetane, the revDSD/junTZ rotational constants agree with experiment with a deviation of 0.5%. The TM approach reduces such an error to 0.3% and, as expected, the inclusion of LR corrections leads to a further improvement, with the mean error and the maximum deviation being 0.12% and 0.15% (for *B*_0_), respectively. For trimethylene sulfide, the initial deviation is similar, 0.56%, which is reduced to 0.15% (on average) by the TM approach and then to 0.14% by TM+LR. In this case, the maximum deviation is 0.25% (and is shown for *A*_0_). In both cases, the application of the TM+LR approach using noncyclic fragments reduces the mean error to 1/4. This suggests that the use, within the TM approach, of noncyclic fragments is reliable and that cyclization effects on the structural parameters are correctly retained from the revDSD/junTZ geometry.

A last comment concerns cyclobutanone, a cyclobutane with one of the CH_2_ moieties substituted by a C=O group. This species is responsible for the maximum error shown in Table 3 for both the revDSD and TM+LR entries. It was observed to behave similarly to inner glycidol, with the angle regulating the position of the oxygen strongly affecting the rotational constants. Analogously to glycidol, it is noted that a change of 2° of the dihedral angle involving the O atom lowers the average error of the rotational constants from 0.37% to 0.07%. In the case of methylenecyclobutane, which has a structure similar to cyclobutanone, this angle is instead already well determined by revDSD/junTZ level and the TM+LR mean error is 0.07%.

For methylenecyclobutane, a partial $r_{e}^{\mathrm{SE}}$ was derived in the present work and is reported in Table 4 (for atom labeling, see Figure 4), where it is compared with the TM+LR structure and the *r*_*s*_ one obtained in ref 42. Only the carbon–carbon bond lengths were determined using the SE approach, while the other parameters were kept fixed at the TM+LR value. The partial $r_{e}^{\mathrm{SE}}$ parameters agree within uncertainties with those at the TM+LR level, with differences of 1–2 mÅ. Instead, the discrepancies between *r*_*s*_ and the equilibrium structure of the present work are more pronounced, these being on the order of 10–12 mÅ. This points out (once again) the unsuitability of the substitution method for deriving equilibrium structures.^5^ Similar conclusions are obtained when inspecting the SE equilibrium structure derived for 2-azetidinone (see Table 4). Deviations between the TM+LR and $r_{e}^{\mathrm{SE}}$ structures are in the order of 1–4 mÅ for distances, and thus well within the estimated uncertainties. The $r_{e}^{\mathrm{SE}}$ angles are affected by small uncertainties and are about 0.05° larger than the TM+LR values. However, if one considers the *r*_*s*_ structure, the deviations are in the order of 10 mÅ for bond lengths and 0.2/0.7° for angles. The C5–N7 bond is somewhat well described by the *r*_*s*_ method with a deviation of 0.7 mÅ. Thus, the TM+LR approach provides geometries, at a very low computational cost, that can reliably be used for the nondeterminable parameters in the semiexperimental structure determinations.

**Figure 4:**
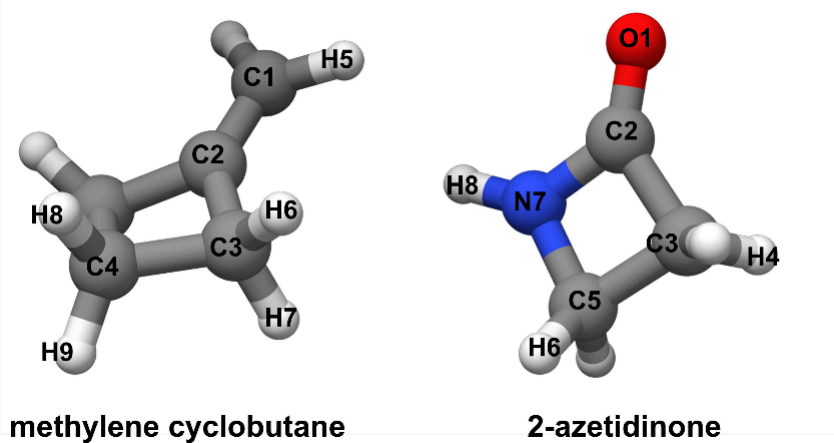
Methylenecyclobutane and 2-azetidinone with atom numbering.

**Table 4:** “Lego-Brick”, Partial $r_{e}^{\mathrm{SE}}$ , and Substitution Structures of Methylenecyclobutane and 2-Azetidinone^a^ (for Atom Labeling, See Figure 4)

	methylenecyclobutane				2-azetidinone		
Parameter	“Lego-brick”	$r_{e}^{\mathrm{SE},}$ ^b^	*r*_*s*_	Parameter	“Lego-brick”	$r_{e}^{\mathrm{SE},}$ ^b^	*r*_*s*_ ^c^
	(TM+LR)	This work	ref 42		(TM+LR)	This work	ref 43
C1C2	1.3282	1.326(1)	1.3383	C1O2	1.2032	1.1996(4)	1.209(3)
C3C4	1.5567	1.554(2)	1.5645	C3C1	1.5399	1.5409(6)	1.554(25)
C1H5	1.0835		1.0901	C5C3	1.5497	1.5467(9)	1.546(13)
C2C3	1.5166	1.5175(1)	1.5100	C5N7	1.4637	1.464(1)	1.476(11)
H6C3	1.0811		1.0965	H4C3	1.0791		-
H7C3	1.0843		1.0934	H6C5	1.0817		-
H8C4	1.0813		1.1029	N7H8	1.0054		0.986(3)
H9C4	1.0800		1.0955	C3C2O1	136.01		136.7(22)
H5C1C2	161.98		163.82	C5C3C2	85.86	85.906(1)	85.2(10)
C1C2C4	121.18		121.81	N7C53	87.28	87.34(2)	87.5(2)
C3C2C1	133.87		133.33	H4C3C2	113.87		
H6C3C2	117.52		117.40	H6C5C2	115.04		
H7C3C2	111.73		112.71	H8N7C5	132.83		132.2(7)
H8C4C2	109.61		113.26	H4C3C2O1	–63.82		
H9C4C2	141.00		138.74	H6C5C3C2	–115.16		
H5C1C2C4	–89.91		–91.48				
C3C2C1H5	–5.91		–7.19				
H6C2C2C1	34.53		38.29				
H7C3C2C1	–92.61		–89.06				

a Bond distances in Å, valence and dihedral angles in degrees (°).

b Standard deviation of the fit: 3.9 × 10^–3^ for methylenecyclobutane and 1.03 × 10^–3^ for 2-azetidinone in units of moment of inertia.

c Based on the Cartesian coordinates reported in ref 43.

Moving to five-atom ring molecules, the statistical uncertainties follow the same trend discussed above for the four-membered cycles, with TM+LR providing a mean absolute error of 0.14%, thus reducing it to 1/4 of the initial revDSD/junTZ value. The similarity between four- and five-membered ring species is also illustrated by the normal distributions of errors shown in panels (a) and (b) of Figure 5, respectively. Indeed, in both panels, it is evident that, starting from the revDSD/junTZ curves (yellow), the TM approach shifts the Gaussian center closer to the *y*-axis, but the corresponding distribution has a larger width. This is then reduced by exploiting the TM+LR scheme, which shows the same standard deviation as the revDSD/junTZ level, but with a more centered Gaussian curve. Figure 5 also points out that the TM+LR rotational constants are, on average, slightly underestimated.

**Figure 5:**
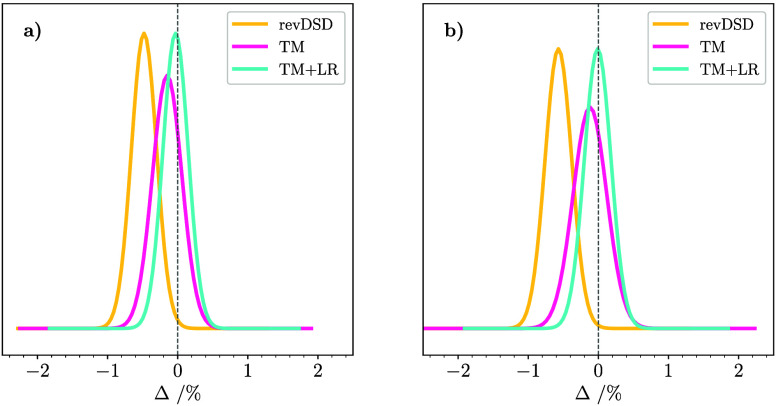
Normal distribution of the errors in the computed rotational constants (*B*_0_^theory,*i*^) in comparison with experimental *B*_0_^exp,*i*^ values for four-membered (panel a) and five-membered (panel b) ring molecules. The levels of theory are revDSD/junTZ (yellow curve), TM (pink curve), and TM+LR (light-blue curve).

Among the 5-membered rings, several experimental structures are available in the literature; for the TM approach, in the present study, we employed those of: furan,^10^ oxazole,^11^ isooxazole,^11^ maleic anhydride,^10^ and cyclopentane.^66^ Only for four species we resorted to open-chain fragments: for tetrahydrofuran and tetrahydrothiophene, we used dimethyl ether or dimethyl sulfide and two methane molecules, in analogy to four-membered rings; a combination of glycine, NH_2_OH, and CH_4_ was employed for the two conformers of cycloserine, while hydantoin was constructed using H_2_CO, NH_3_, and glycine.

The accuracy of the rotational constants obtained using the TM+LR approach allows to have some insights on the tetrahydrofuran structure. This molecule has three possible conformations with different symmetry as shown in Figure S4 of the SI. The conformer with *C*_2*v*_ symmetry is a transition state, as already reported in the literature.^71,72^ The other two conformations are very close in energy, with the *C_s_* bent structure being only 0.04 kJ/mol lower in energy than the *C*_2_ twisted form, if equilibrium revDSD/junTZ energies are considered. This stability order is reversed when the zero-point vibrational correction (from B3/junDZ calculations) is included, with the twisted structure becoming lower in energy by ∼0.6 kJ/mol. In passing we note that the twisted conformation was found to be the most stable by CCSD(T)/CBS+CV energetic estimates^73^ and experimental analyses,^51^ in disagreement with a recent theoretical work.^71^ As the two conformations lie very close in energy and interact through a ring puckering motion, it is difficult to establish, solely on the basis of energetic arguments, if there is a predominantly observed conformer in experimental setups. For this reason, we have derived the TM+LR structure for both the bent/envelope and the twisted structures; the results are shown in Figure S4 of the SI. At the revDSD/junTZ level, the rotational constants of these conformations show a mean deviation of 0.8% and 0.6%, respectively, from experiment. Therefore, this level of theory is not able to discriminate between the two structures. However, exploitation of the TM+LR approach reveals that the twisted (*C*_2_) structure yields results that are in better agreement with experiment, with a mean absolute error for the rotational constants of 0.26%. Instead, for the *C_s_* structure, the TM+LR approach leads to no improvement at all, the mean deviation remaining as large as 0.8%. Thus, these results suggest that the twisted geometry corresponds to the most stable structure of the molecule. We note that the comparison of the rotational constants was carried out using the lowest energy tunneling sublevel of ref 51, but our conclusions remain unchanged even if one considers the other levels. The *C*_2_ symmetry conformer is the most stable form also in the case of tetrahydrothiophene, as already established in the literature;^52^ the average error on the rotational constants is 0.2%, in analogy to its oxygen counterpart.

Another aspect that deserves to be addressed concerns the employment of a partial $r_{e}^{\mathrm{SE}}$ within the TM+LR approach. For methylenecyclopentane, cyclopentane was used to template the carbon backbone. However, for the latter, only three C–C bond lengths were determined.^66^ Even if partial, the effect of the $r_{e}^{\mathrm{SE}}$ parameters within the TM approach is relevant, with the mean error lowering from 0.48% (revDSD/junTZ) to 0.15% (TM+LR).

Hydantoin and 2,5-oxazolidinedione were observed in laser ablation experiments as cyclization products of hydantoic acid.^53^ Therefore, ref 53 offers the opportunity of investigate the applicability of the TM+LR approach not only to a flexible chain (hydantoic acid) but also to its cyclic derivative (hydantoin), which share common fragments. In particular, the C5-I, C5-II and C7-II conformers of hydantoic acid were included in the data set, while the C7-I form was excluded due to nonreliable VPT2 results (here we recall that reliable vibrational corrections to rotational constants are mandatory for a meaningful comparison with experiment). As mentioned above, the fragments employed to template the hydantoic acid are the same as hydantoin, namely H_2_CO, NH_3_, and glycine. On average, TM+LR rotational constants deviate from the experimental counterpart by 0.35% for hydantoin, 0.15% for C5-I and C7-II, and 0.28% for C5-II. For hydantoin, the larger deviation is due to a large discrepancy on the *A*_0_ constant, while *B*_0_ and *C*_0_ show an accuracy better than 0.2%. While the results obtained for the hydantoic acid are encouraging, a more detailed study on open-chain species is needed. However, this requires an extension of the semiexperimental data set to relevant fragments such as methanol, ethanol, and methylamine. Although structurally simple, these molecules have internal motions that complicate their spectral analysis, which leads to rotational constants that are poorly defined.

### Larger Ring Molecules

This part of the data set offers useful insights into the application of the TM+LR approach to constitutional isomers and to species with a large conformational space. Starting from the former case, toluene, 3-methylene-1,4-cyclohexadiene, and spiro­[2,4]­hepta-4,6-diene belong to the C_7_H_8_ family and show consistent results, with the TM+LR approach reducing the deviation of the rotational constants from 0.4% (revDSD/junTZ) to less than 0.1%. While these species show a certain degree of structural rigidity, an increased degree of flexibility is represented by caffeic acid, which has eight different geometrical isomers. These species have been templated using benzene, water, and acrylic acid. For the *cis* and *trans* forms of acrylic acid, a partial $r_{e}^{\mathrm{SE}}$ structure has been determined for the first time in this work and is reported in Table S1 of the SI. Across the eight structures, the mean deviation of the revDSD/junTZ rotational constants from experiment is 0.50–0.56%. Exploitation of the TM+LR approach leads to major improvements, with average discrepancy lowering to 0.10–0.12% and a maximum deviation of 0.27%. This suggests once again that even if only a partial $r_{e}^{\mathrm{SE}}$ is available, its use within the TM+LR scheme can lead to significant improvements.

The most flexible species of this and the entire data set are aspirin and serotonin. These have two and three conformers, respectively, and provide the occasion to point out the challenge arising from the application of the TM approach whenever there is a lack of suitable fragments because of the dimensions of the target system. The TM strategy employed for these molecules is shown in Figure 6. Aspirin was considered as a benzene molecule functionalized with formic acid and methyl formate. To the latter, an additional CH_4_ molecule is linked to reproduce the terminal CH_3_ group. For serotonin, the difficulty is the description of the lateral −CH_2_CH_2_NH_2_ chain. This bond pattern is not well reproduced by isolated CH_4_ fragments, and a way-out was offered by the use of propanal for the central CH_2_CH_2_ chain. Overall, the results are satisfactory, with both conformers of aspirin being predicted with Δ_*k*_ below 0.2% at the TM+LR level. For serotonin, the approach leads to even lower deviations, with seven rotational constants being predicted with an error smaller than 0.1% and the remaining two with a deviation of 0.1%.

**Figure 6:**
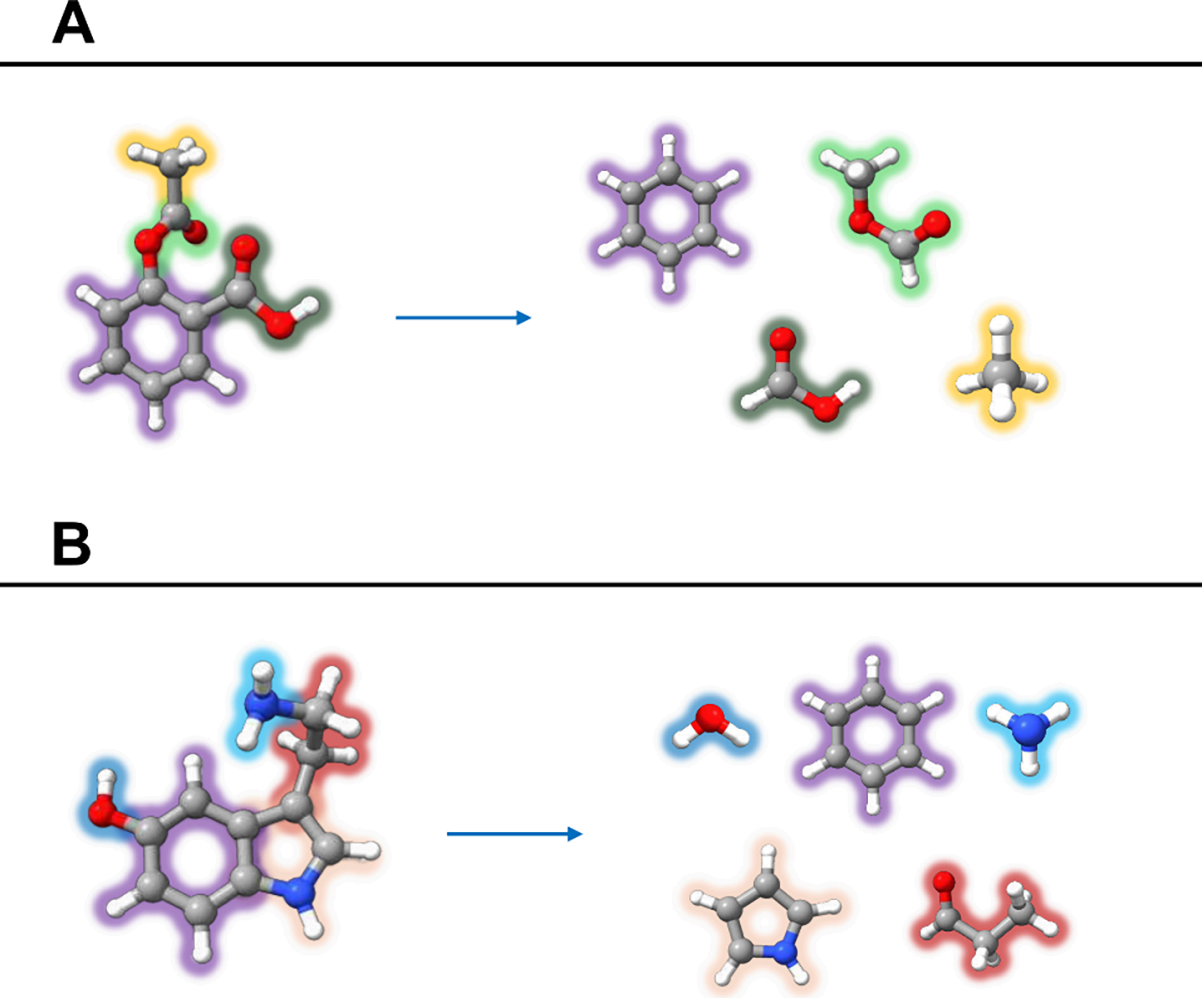
Fragments employed within the TM approach applied to aspirin (panel A) and serotonin (panel B).

The complete set of statistics for six-membered ring molecules and bicyclic compounds is graphically represented in Figure 7, where the improvement offered by TM and TM+LR compared to revDSD/junTZ is noticeable. In particular, the TM+LR normal distribution appears narrow and nearly centered in the origin.

**Figure 7:**
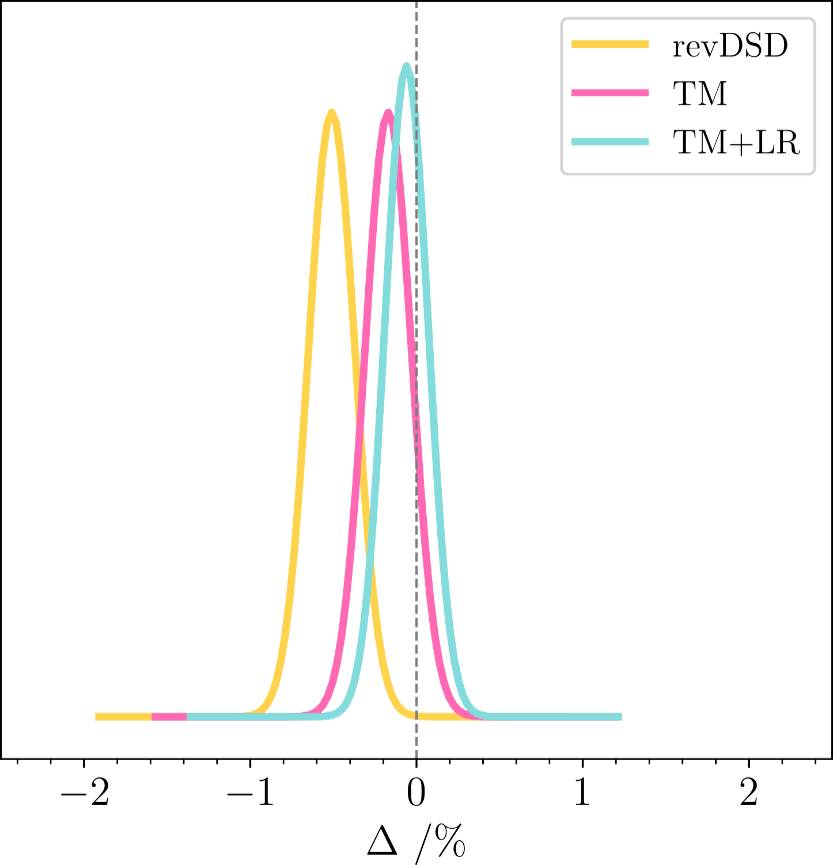
Normal distribution of the errors in the computed rotational constants (*B*_0_^theory,*i*^) in comparison with experimental *B*_0_^exp,*i*^ values for large cyclic species (six or more atoms in the ring) and linear chains. The levels of theory are revDSD/junTZ (yellow curve), TM (pink curve), and TM+LR (light-blue curve).

## Conclusions

The application of the “Lego-brick” (TM+LR) approach to strained rings and cyclic molecules with functionalized chains led to the determination of a systematic trend in the estimate of the rotational constants. The application of the TM approach tends to give a slight worsening of the results in terms of standard deviation, but to an improvement of the mean deviation. This is further lowered by incorporating the LR corrections (thus leading to the TM+LR scheme), the standard deviation returning to the revDSD/junTZ values.

Overall, the rotational constants resulting from the TM+LR approach show improvements with respect to the initial revDSD/junTZ values, with a typical reduction to 1/4 or better, of the initial mean errors. This often leads to an accuracy better than 0.1% with respect to experiment. Whenever this typical accuracy of the TM+LR approach is not obtained, the reason can be traced back to the sensitivity of the rotational constants to specific structural parameters (usually dihedral angles), as shown for glycidol and cyclobutanone. Still, even in these cases, the TM+LR model provides a sensible improvement with respect to revDSD/junTZ values without any additional computational cost and offers a good starting point for the derivation of accurate equilibrium structures via the semiexperimental approach, as shown for 2-azetidinone and methylenecyclobutane.

Focusing on the application of the TM approach, this work has provided some insights into the exploitation of partial $r_{e}^{\mathrm{SE}}$ structures for the templating procedure, as in the case of methylenecyclopentane and caffeic acid. The results seem to be very promising, even if the TM approach is only applied to a subset of bond lengths of the target molecule. Furthermore, the possibility of using open-chain fragments for templating rings and bicyclic structures was explored, with solid results being obtained for the cases considered. Still, the lack of fragments might prevent the application of the “Lego-brick” approach and a possible way-out proposed in this work is a strategy relying on envisaging additional fragmentation of a given fragment, that is a fragment is seen as a smaller fragment (subfragment) functionalized with an additional subfragment.

In the present work, we mainly relied on a consistent data set of SE equilibrium geometries available in the literature.^11^ The $r_{e}^{\mathrm{SE}}$ geometries collected in ref 11 were obtained using different levels of theory for the calculation of the required vibrational corrections, ranging from fc-CCSD­(T)/cc-pVTZ (fc denoting the frozen-core approximation) to revDSD/junTZ. Previous studies have shown that DFT- and CC-based vibrational corrections lead to comparable $r_{e}^{\mathrm{SE}}$ structures,^9,10^ especially when double-hybrid functionals are employed. An example in this respect is offered by NH_3_, for which a high-quality $r_{e}^{\mathrm{SE}}$ based on ae-CCSD­(T)/cc-pwCVQZ (ae denoting all electrons being correlated) vibrational corrections has been reported in ref 74. This $r_{e}^{\mathrm{SE}}$ sets the N–H distance to 1.0106(4) Å and the HNH angle to 106.83(9)°. Using double-hybrid DFT vibrational corrections,^9^ these SE equilibrium parameters are determined to be 1.0111(1) Å and 106.93(2)°, respectively, thus showing a discrepancy of 0.5 mÅ for N–H and 0.1° for HNH. Therefore, the differences are marginal and the two $r_{e}^{\mathrm{SE}}$ structures agree within the given uncertainties. Moving to larger and cyclic species, analogously, the differences between the SE equilibrium structures derived using vibrational corrections at different levels are, in the vast majority of the cases, well within the quoted uncertainties. It is worthwhile noting that recent works on pyridine, pyridazine, and pyridine,^75–77^ which can be considered representative molecules in this respect, have shown that the employment of a large set of isotopologues combined with vibrational corrections based on CC theory leads to a improvement of the statistical uncertainties of the structural parameters, which are however still in very good agreement with those derived from SE fitting procedures employing DFT vibrational corrections. The reader is referred to, for example, Table 4 of refs 75 and 76.

Overall, in view of the marginal modifications in the geometrical parameters resulting from the use of different-level vibrational corrections in the SE procedure, it is expected that improvements in the $r_{e}^{\mathrm{SE}}$ based on the quality of the vibrational corrections lead only to marginal modifications in the results of the TM+LR approach. Instead, the accuracy of the latter tends to be dominated by the uncertainty affecting dihedral angles and, more generally, by those parameters that cannot be corrected using either the TM or LR approach, provided that the $r_{e}^{\mathrm{SE}}$ structures employed are not affected by a bias resulting, for instance, from an unbalanced set of isotopic species.

Future developments of the TM+LR framework will focus on open-chain species and will aim at deriving correction terms for dihedral angles connecting molecular fragments. This will require the derivation of new semiexperimental equilibrium structures for spectroscopically complex species, characterized by internal rotations or large amplitude motions, also implying the need of high-quality structures of reference.

## Supplementary Material



## Notes

Detailed representation of the TM approach for each molecule of the data set; structures of tetrahydrofuran with results from TM+LR approach; semiexperimental structure of *trans*- and *cis*-acrylic acid; and revDSD/junTZ, TM, and TM+LR structures of the species of the benchmark data set using the Z-matrix format (PDF)

## Notes

This work is in loving memory of John F. Stanton. His scientific work will live on in the pages of hundreds of papers, and its importance will never be forgotten.
