# Supplementary material for: Assessing the Limits of the “Lego-Brick” Approach: Equilibrium Structures of Strained and Flexible Cyclic Molecules
Source: J Phys Chem A. 2026 Apr 15;130(17):3403–15. doi: 10.1021/acs.jpca.6c00650 (PMC13137258; doi:10.1021/acs.jpca.6c00650)
Supplement: Supplementary file 1 [file jp6c00650_si_001.pdf]

Supporting Information for "Assessing the Limits of the "Lego-Brick" Approach: Equilibrium Structures of Strained and Flexible Cyclic Molecules"

Silvia Alessandrini,* Alessandra Savarese, Mattia Melosso, Luca Bizzocchi, and Cristina Puzzarini*

Dipartimento di Chimica "Giacomo Ciamician", Università di Bologna, Via P. Gobetti 85, I-40129 Bologna, Italy

E-mail: silvia.alessandrini7@unibo.it; cristina.puzzarini@unibo.it

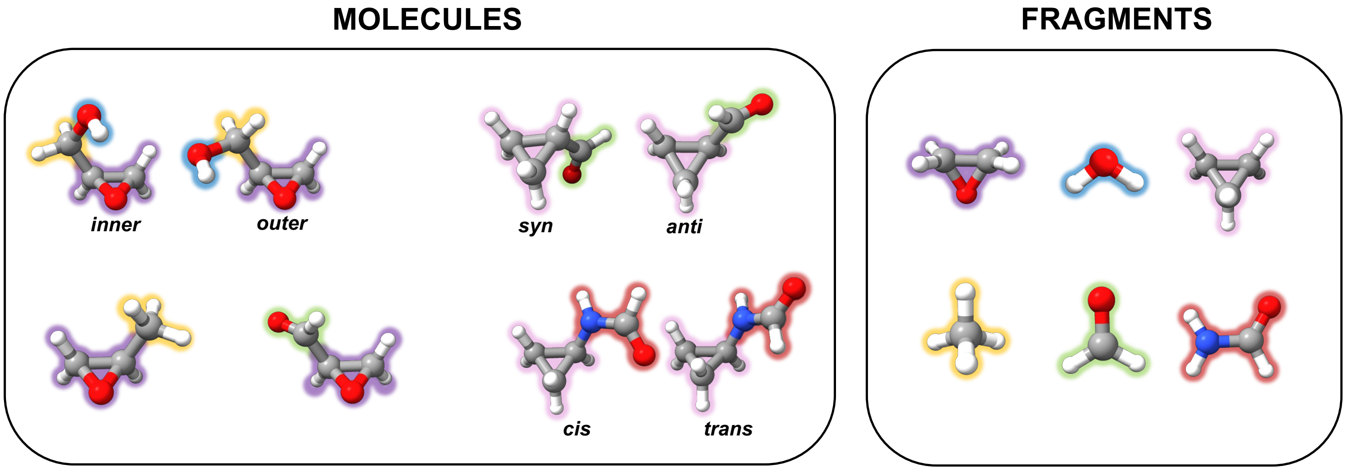

Figure S1: Illustration of the TM approach for each three-membered ring molecule studied in this work, with fragments.

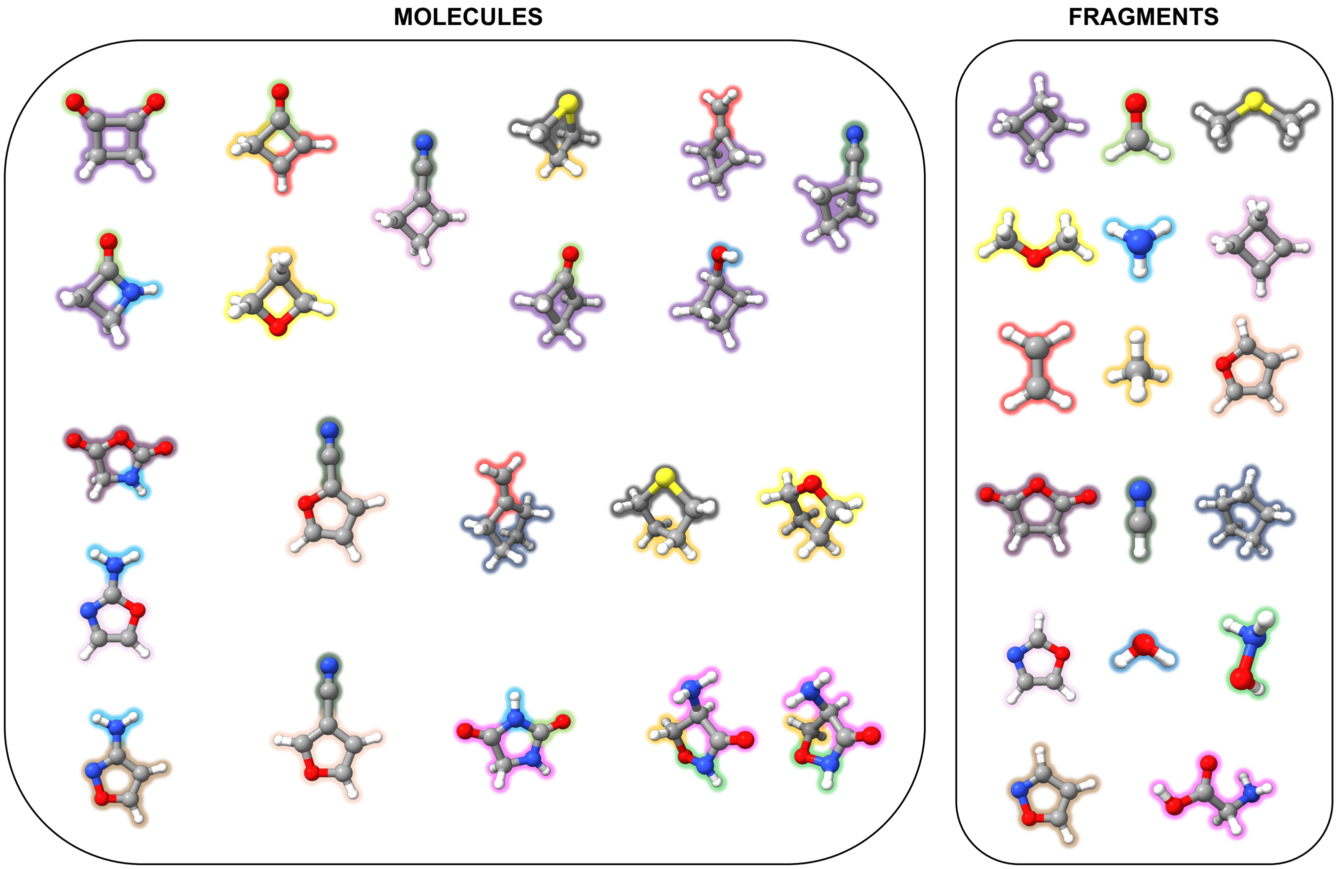

Figure S2: Illustration of the TM approach for the four- and five-membered ring molecules studied in this work, with fragments.

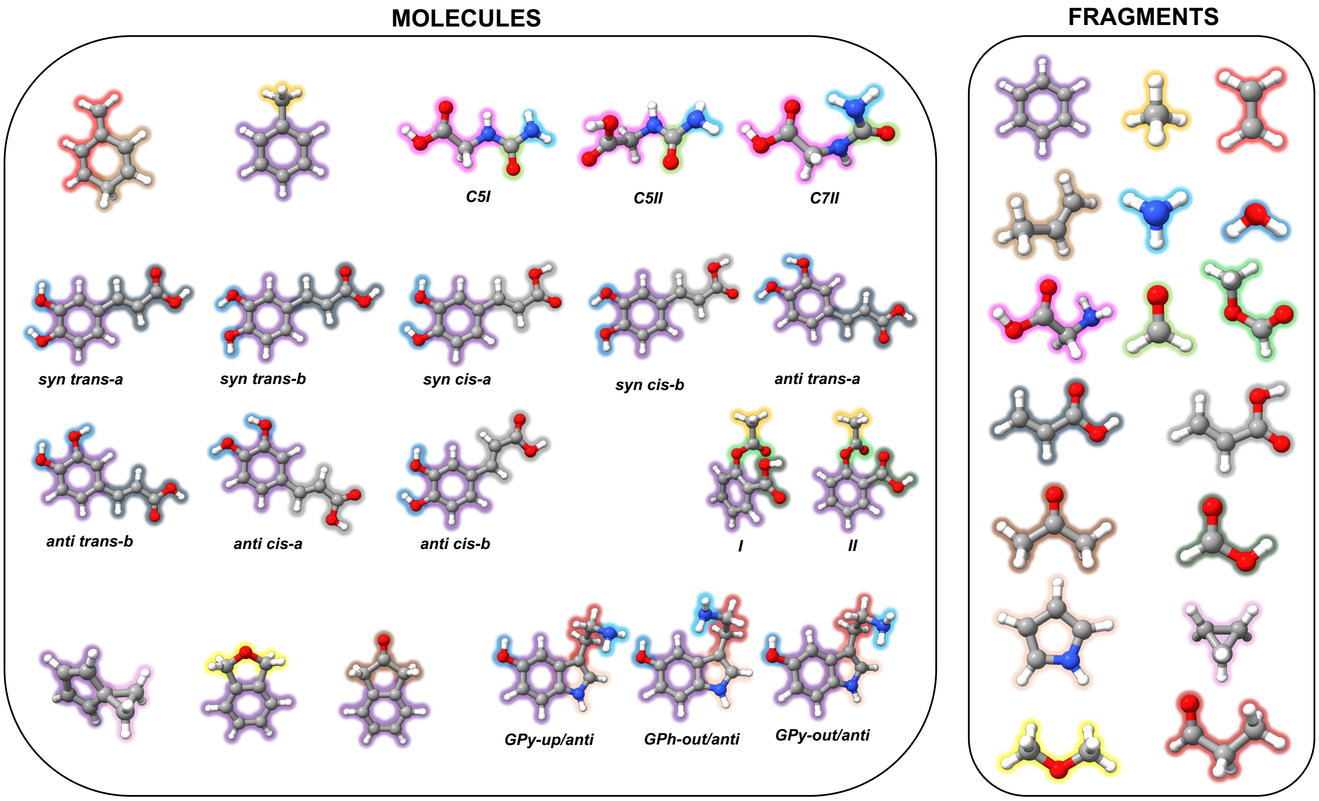

Figure S3: Illustration of the TM approach for the six-membered ring molecules, bicyclic structures and open-chain species studied in this work, with fragments.

TWISTED (C_2)

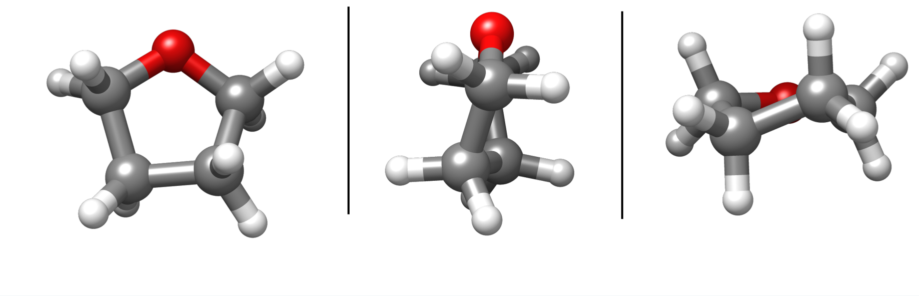

	Exp.	revDSD/ junTZ	TM+LR
A_0	7096.770(2)	-0.66%	0.55%
B_0	6976.371(2)	-0.42%	-0.07%
C_0	4008.219(2)	-0.68%	0.15%

ENVELOPE (C_v)

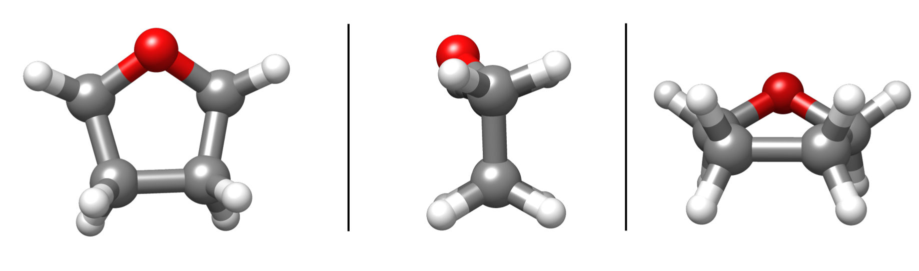

	Exp.	revDSD/ junTZ	TM+LR
A_0	7096.770(2)	-0.11%	1.11%
B_0	6976.371(2)	-1.38%	-1.06%
C_0	4008.219(2)	-1.06%	-0.25%

PLANAR TRANSITION STATE (C_{2v})

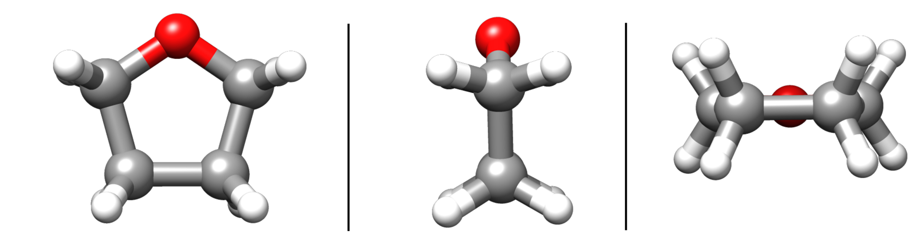

Figure S4: The two conformations of tetrahydrofuran with experimental rotational constants compared to the revDSD/junTZ and TM+LR results. The deviations reported are Δ_k in %.

Table S1: Semi-experimental equilibrium structure[a] of s-*cis*- and s-*trans*-acrylic acid compared with the revDSD/junTZ and TM+LR counterparts.

Parameter	s-*cis*-acrylic acid			s-*trans*-acrylic		
	revDSD /junTZ	Lego-brick (TM+LR)	r^{SE}	revDSD /junTZ	"Lego-brick" (TM+LR)	r^{SE}
C1O2	1.2090	1.2056		1.2106	1.2070	
C1O3	1.3550	1.3510	1.3481(4)	1.3546	1.3521	1.3494(5)
O3C1O2	122.75	122.77		122.50	122.45	
H4C3	0.9685	0.9648	0.9611(4)	0.9678	0.9641	0.9616(6)
H4O3C1	106.23	106.30		105.84	105.84	
C1C5	1.4824	1.4782	1.4794(3)	1.4785	1.4755	1.4738(5)
C5C1O2	126.09	125.77	125.74(1)	123.88	123.70	123.82(4)
C6C5	1.3333	1.3321	1.3321(4)	1.3344	1.3329	1.3316(12)
C6C5C1	120.40	120.07	120.17(3)	123.80	123.60	123.66(10)
H7C5	1.0824	1.0800		1.0823	1.0796	
H7C5C1	117.17	117.33		114.09	114.07	
H8C6	1.0834	1.0804		1.0822	1.0794	
H8C6C5	120.19	120.03		121.21	121.10	
H9C6	1.0822	1.0795		1.0827	1.0798	
H9C6C5	121.29	121.27		120.81	120.77	

[a] Bond distances in Å, angles in degrees. The non-determinable parameters of r_e^{SE} structures have been kept fixed at the corresponding TM+LR values. The standard deviation of the fit is 7.5×10^{-4} and 1.21×10^{-3} in units of moment of inertia for the *cis* and *trans* forms, respectively.
